# Physical Trauma Patients with Symptoms of an Acute and Posttraumatic Stress Disorder: Protocol for an Observational Prospective Cohort Study

**DOI:** 10.2196/resprot.9006

**Published:** 2018-03-29

**Authors:** Eva Visser, Taco Gosens, Brenda Den Oudsten, Jolanda De Vries

**Affiliations:** ^1^ Trauma TopCare Elisabeth-TweeSteden Hospital Tilburg Netherlands; ^2^ Department of Orthopaedics Elisabeth-TweeSteden Hospital Tilburg Netherlands; ^3^ Center of Research on Psychological and Somatic Disorders Department of Medical and Clinical Psychology Tilburg University Tilburg Netherlands; ^4^ Department of Medical Psychology Elisabeth-TweeSteden Hospital Tilburg Netherlands

**Keywords:** acute stress disorder, posttraumatic stress disorder, PTSD, ASD, trauma, injury, observational study, qualitative study, focus groups

## Abstract

**Background:**

Injury, medical treatment, and rehabilitation can have major impacts on patients’ wellbeing. About 25-33% of the patients experience an acute stress disorder (ASD) or a posttraumatic stress disorder (PTSD) after injury. ASD is a relatively new diagnosis. Therefore, knowledge about patients’ experiences, the course of ASD and PTSD, and who is at risk for developing ASD or PTSD is lacking.

**Objective:**

The aims of this multi-method study are to explore patients’ experiences with injury (and their care) using a focus group study. Then, in the observational study, different courses of ASD, PTSD, and quality of life will be examined. In addition, this study will examine if these courses could be characterized by socio-demographic, clinical, and psychological variables. Consequently, a risk profile will be developed to determine which patients are at risk for developing ASD or PTSD during the 12 months after injury.

**Methods:**

Trauma patients treated in the shock room (in 2015) of the Elisabeth-TweeSteden Hospital will share their experiences with injury in the focus group study. Open, axial, and selective coding will be used to analyze the data. Concerning the observational study, patients treated in the shock room (during 2016 and 2017, Elisabeth-TweeSteden Hospital and Erasmus Medical Centre) will be asked to participate. The inclusion period is 12 months. Participants will complete the Impact of Event Scale-Revised, MINI-plus, the Hospital Anxiety and Depression Scale, and the World Health Organization Quality of Life-BREF after inclusion and at 3, 6, 9, and 12 months after injury. The NEO-Five Factor Inventory and the State-Trait Anxiety Inventory-Trait are completed after inclusion only. Repeated measures of latent class analysis and linear mixed models will be used to examine the research aims.

**Results:**

This project was funded in August 2015 by ZonMw. The results of the focus group study are expected in the first trimester of 2018. With regard to the observational study, recruitment is currently underway. Data collection will be completed in November 2018. The first results will be expected in the first trimester of 2019.

**Conclusions:**

This is the first multi-method study in trauma patients that examines patients’ experiences (qualitative design) as well as psychological disorders (observational prospective). This study will contribute to necessary information on psychological consequences after injury. Moreover, it provides knowledge about which patients to include in future psychological intervention research. Finally, awareness in clinicians about the psychological consequences can be created, so they are able to act more effectively to provide patient-oriented care.

**Trial Registration:**

Netherlands Trial Registry NTR6258; http://www.trialregister.nl/trialreg/admin/rctview.asp?TC=6258 (Archived by WebCite at http://www.webcitation.org/6xSCiO1bS)

## Introduction

Due to registration and implementation of specialized trauma care, the quality of medical treatment has been improved and survivorship has been increased [1-6]. Trauma is related to physical disabilities (eg, pain, fatigue and impaired wound healing), acute stress disorder (ASD), posttraumatic stress disorder (PTSD), and psychological distress [7-13]. Moreover, trauma patients experience an impaired quality of life (QOL) compared to the general population [14-20].

About 25% of trauma patients have subsyndromal ASD during hospitalization and about 30% had PTSD one month after injury [8,21]. Six months after injury, 49% showed a delayed onset of PTSD. This percentage decreased to 20% at 24 months after injury. A recent systematic review showed that patients diagnosed with ASD had a higher risk of developing PTSD [8]. However, the prevalence rate of patients with ASD who develop PTSD is unknown. Diagnostic criteria for ASD and PTSD are similar; however, dissociative symptoms (eg, depersonalization, derealization, and dissociative amnesia) are only emphasized in ASD and not in PTSD. Moreover, ASD can only be diagnosed within the first month after trauma and last for less than a month, while PTSD symptoms persist for at least one month after injury [22]. PTSD symptoms may begin either after trauma or months or years afterwards [23].

In addition to QOL, PTSD, anxiety, and depression are most frequently examined after injury [15-20]. However, information about ASD is scarce. The existing studies of ASD and PTSD are often cross-sectional. Moreover, in the case of an observational prospective design, examination of PTSD is limited to only several months after trauma. One or several measurements are needed to examine patients’ psychological recovery shortly after injury. Important information about the courses of ASD and PTSD (ie, main scores of onset and development, such as the stability of symptom severity over time) and patients’ characteristics is lacking [8,24,25]. More specifically, it is unknown if and in what way patients’ experiences with injury and treatment, for instance, in the shock room, contribute to psychological consequences. Moreover, factors related to communication between medical staff and patient, treatment of injury, and environment are not known. Gaining information about the development of ASD and PTSD and their sustaining risk factors will increase the quality of care because patients at risk can be offered psychological treatment, thereby preventing the development of psychological disorders, such as ASD and PTSD. Health care providers with the knowledge of medical and psychological consequences after trauma can better anticipate patients’ needs so that patient-centered care can be provided.

This multi-method study consists of a focus group study and an observational prospective study. The ultimate goal of this multi-method study is to provide valuable insight into the severity of psychological consequences, including ASD and PTSD, and the need for a psychological intervention study to prevent PTSD. First, focus groups are held to examine patients’ experiences with injury (and their care). In this way, potential factors related to the development of psychological problems (eg, depressive symptoms) and disorders (eg, anxiety, ASD, and PTSD) can be obtained and taken into account for the observational study (aim 1). Subsequently, an aim of the observational study is to examine the courses of ASD and PTSD (aim 2). In addition, it will be examined which socio-demographic (ie, sex, age, marital status, and education level), clinical (ie, type of trauma, Injury Severity Score (ISS), Glasgow Coma Score, being hospitalized, being treated on the intensive care unit, complications during treatment, and treatment by a medical psychologist or psychiatrist), and psychological variables (eg, anxiety, depressive symptoms, and personality) characterize the courses of ASD and PTSD. Subsequently, a risk profile will be developed to determine which patients are at risk for ASD and/or PTSD (aim 3). Finally, to study the effect(s) of the natural course of ASD symptoms on the development of PTSD, anxiety and depressive symptoms, and QOL across time will be analyzed (aim 4).

## Methods

### Design

**Figure 1:**
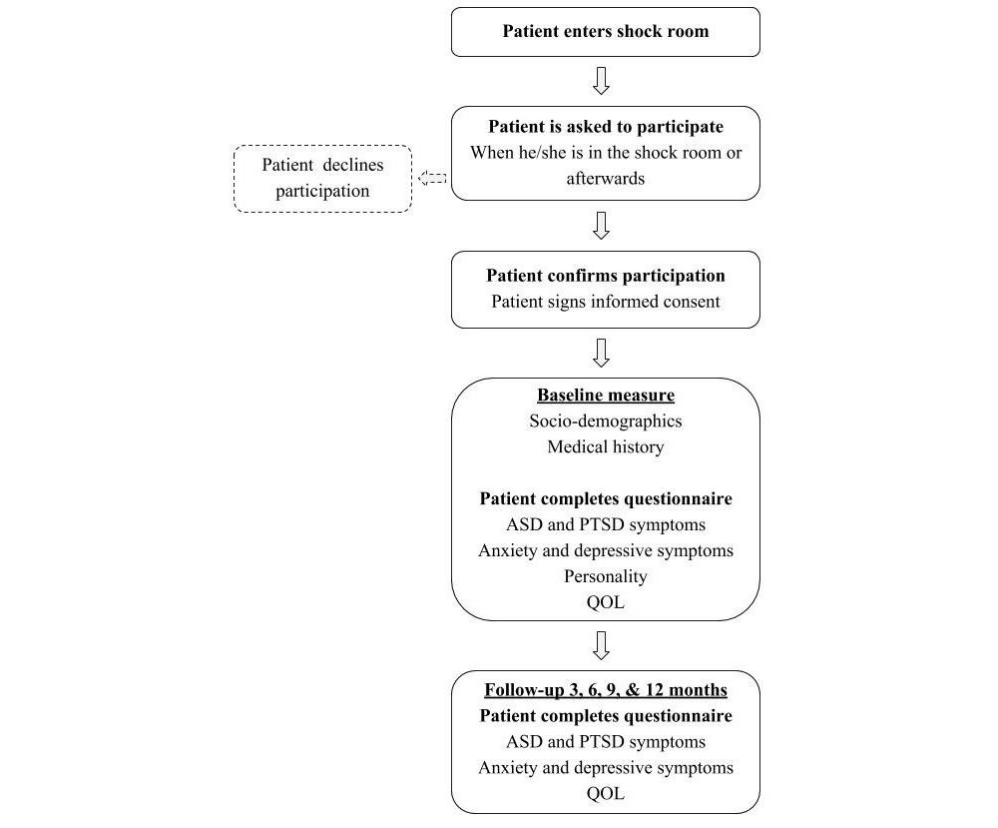
Flowchart of the study design. ASD: acute stress disorder; IC: informed consent; PTSD: Posttraumatic stress disorder; QOL: quality of life.

First, using a qualitative focus group study design, patients’ perspectives on the injury, treatment in the shock room and hospital, and rehabilitation are explored. A focus group is a commonly used method of qualitative research as it is a valid and reliable technique. Moreover, focus groups facilitate the in-depth exploration of a person’s perspective through group interaction. Participants can be triggered by a comment from another participant [26,27], and by the concept of sharing and comparing [28]. Then, as an extension of the focus group study, the observational prospective cohort study will examine ASD, PTSD, anxiety and depressive symptoms, and QOL. This will be assessed up to one year after treatment for physical trauma. A flow diagram of the observational study design and the main procedures that patients will undergo during the course of the observational study are shown in Figure 1.

### Participants and Centers of Recruitment

Trauma patients treated in the shock room in 2015 of the Elisabeth-TweeSteden Hospital are asked to participate in the focus group study. A shock room is situated at the Emergency Department and is reserved for physical trauma patients (ie, all types of injury) with a potentially life-threatening situation.

Concerning the observational study, all adult patients who have been in the shock room at the Emergency Departments of the Elisabeth-TweeSteden Hospital (Tilburg) or the Erasmus Medical Centre (Rotterdam, The Netherlands) are asked to participate. The inclusion period is about 12 months after the start in November 2016.

### Sample Size Calculation

This project is exploratory in nature. Moreover, the focus is on examining the stability of results. The sample size was calculated only for the observational study. According to the Dutch trauma registry, the shock room admission was about N=1440 in 2013 and N=986 in 2016 in the Elisabeth-TweeSteden Hospital. Using a mean of these admission numbers of (N=1213, alpha=0.05, beta=0.80, and effect size=0.4), it was estimated that N=300 would be sufficient. This was also based on Monte Carlo simulations [29].

### Inclusion and Exclusion Criteria

In order to be eligible to participate, patients (1) are treated in the shock room and (2) are aged 18 or older. Patients are excluded from participation in case of (1) severe traumatic brain injury (ie, Glasgow Coma Score ≤ 8), (2) dementia, or (3) insufficient knowledge of the Dutch language (verbal and writing). These criteria are used in the focus group and the study observational study.

### Study Procedures

#### Focus Groups

Trauma patients who were treated in the shock room of the Elisabeth-TweeSteden Hospital during 2015 were asked to participate in a qualitative focus group study. Patients were divided into 3 groups: (1) patients who went home after treatment in shock room (no hospitalization) or, in case of hospitalization, they had an ISS of less than 16; (2) ISS equal or higher than 16, and (3) mild or moderate traumatic brain injury (Glasgow Come Score > 8). Six to 10 patients were invited to participate in each group. To obtain a representative sample of the trauma population, the division into groups was based on type of injury, sex, and age. The purposive sampling method was used [26,27].

The focus group meetings took place in a conference room at the Elisabeth-TweeSteden Hospital. Each focus group was guided by a moderator and an assistant. The patients were asked to share their experiences by answering the main question, “What experience related to your injury impressed you the most?” Their experiences were clustered on a flipchart on the basis of the trauma procedure: (1) moment of injury, (2) treatment in the ambulance or the trauma helicopter, (3) treatment in the shock room, (4) hospital stay, (5) moment of discharge, and (6) period after discharge and/or rehabilitation. Finally, another main question, “In what way did you need and received psychological treatment?”, was discussed. At the end of each focus group, participants were asked to complete questions about their socio-demographic status (ie, age, sex, marital status, and education level). In addition, they completed the Impact of Event Scale revised (IES-R) for PTSD and the Hospital Anxiety and Depression Scale (HADS) for anxiety and depressive symptoms. All focus groups had the same structure and were audio-recorded. The duration of the meeting was about 90 minutes.

#### Observational Study

The emergency doctor or the resident will ask patients to participate in this study as soon as they can talk and are lucid. If the emergency doctor or the resident is not able to ask the patient to participate (eg, due to transferring the patient to another department in the hospital), the researcher will ask the patient as soon as possible to participate in this study. The researcher will check medical records to see whether there are patients that have not yet been asked to participate in the study.

Patients will sign two informed consents. First, in the emergency department (after being treated in the shock room and being informed by the doctor). Then 1-5 days later, the patient will be asked to confirm participation again to make sure that they have sufficient time to consider participation in the study. In the case of a patient who is unconscious, the patient will be informed by the researcher and asked to participate as soon as the patient is lucid. If a patient declines participation by not signing the second informed consent, all obtained information will be destroyed.

After confirming participation, the patient will complete a questionnaire on socio-demographic questions, ASD and PTSD, anxiety and depressive symptoms, personality, and QOL at the first time-point (ie, baseline). Clinical information will be retrieved from patients’ medical records. The measurement points are at inclusion, 3, 6, 9, and 12 months after injury (see Figure 1). 

### Data Collection

#### Focus Groups

The topic of the interviews are focused on patients’ experiences with the traumatic event (see Study Procedures). In addition, participants were asked to complete socio-demographic questions, the IES-R and the HADS. All focus groups have the same structure and are audio-recorded. The recorded focus groups are transcribed verbatim [26,27].

In case of observed severe symptoms of ASD or PTSD during focus group sessions, the treating physician was informed. The doctor could refer the patient for a consult with a psychologist in the department of Medical Psychology at the Elisabeth-TweeSteden Hospital who is specialized in psychological treatment after injury.

#### Observational Study

Data for the observational study will be collected using a structured interview—MINI-plus for ASD and PTSD—as well as self-report questionnaires: (1) the IES-R for ASD and PTSD, (2) HADS, (3) NEO Five-Factor Inventory (NEO-FFI) and the State Trait Anxiety Inventory (STAI) - Trait scale for personality, and (4) World Health Organization Quality of Life assessment instrument-Bref (WHOQOL-Bref) for QOL. All outcome measures will be assessed after treatment in the shock room (baseline), 3, 6, 9, and 12 months after injury. However, ASD and personality will only be measured at baseline (see Table 1).

##### Acute Stress Disorder and Post Traumatic Stress Disorder

The MINI-Plus [22] and the IES-R [30] assess ASD and PTSD symptoms. Since both instruments are often used (together) in clinical practice, we will use both in the current study.

The MINI-Plus is a short structured interview based on the Diagnostic and Statistical Manual of Mental Disorders (DSM-5) and it will be used to assess ASD and PTSD symptoms [22]. The items are dichotomous because symptoms are present or absent. The DSM-5 is a classification of mental disorders with associated criteria designed to facilitate more reliable diagnoses of these disorders compared to the DSM-IV. It is a standard reference for clinical practice in the field of mental health [22]. For diagnostic criteria for ASD, see Multimedia Appendix 1 and for PTSD, see Multimedia Appendix 2.

The IES-R is a self-report questionnaire to assess symptom severity of ASD and PTSD [30]. It consists of 15 items which measure intrusive re-experiences of the injury and avoidance of injury-related stimuli. The respondent states whether the content of each statement was present during the past 7 days. A 4-point Likert scale will be used ranging from 0 (*not at all*) to 5 (*often*). The cut-off score for a probable diagnosis of PTSD is ≥ 33 and have good diagnostic accuracy [31,32]. The IES-R has good psychometric properties [32] and the Dutch translation of the IES-R has been found to be valid and reliable [33].

##### Anxiety and Depressive symptoms

The HADS measures anxiety and depressive symptoms [34]. It is a generic questionnaire measuring levels of anxiety (7 items) and depression (7 items) with a 4-point rating scale ranging from 0 (*not at all*) to 3 (*very much*). Subscale values ≥11 for one of the subgroups are observed as an indication for a psychological disorder, as this cut-off score provides the lowest proportion of false positives (5% for anxiety and 1% for depression) [34]. The questionnaire is shown to be reliable and valid [34].

**Table 1:** Overview of self-report questionnaires

Study and related questionnaires		Domain	Outcome measures	Time point for retrieval
**Focus group study**				
	Patients’ experiences	N/A^a^	Primary outcome	N/A
	IES-R^b^	PTSD^c^	Secondary outcome	Shortly after meeting
	HADS^d^	Anxiety Depressive symptoms	Secondary outcome	Shortly after meeting
	Sociodemographic questions	Educational level Living situation Paid job	Secondary outcome	Shortly after meeting
**Observational study**				
	MINI-Plus	ASD^e^ PTSD	Primary outcome	Baseline 3 months 6 months 9 months 12 months
	IES-R	ASD PTSD	Primary outcome	Baseline 3 months 6 months 9 months 12 months
	HADS	Anxiety Depressive symptoms	Secondary outcome	Baseline 3 months 6 months 9 months 12 months
	NEO-FFI^f^	Personality	Secondary outcome	Baseline
	STAI^g^-Trait	Personality	Secondary outcome	Baseline
	WHOQOL-Bref^h^	QOL^i^	Secondary outcome	Baseline 3 months 6 months 9 months 12 months

^a^N/A: Not applicable.

^b^IES-R: Impact of Event Scale-Revised.

^c^PTSD: posttraumatic stress disorder.

^d^HADS: Hospital Anxiety and Depression Scale.

^e^ASD: acute stress disorder.

^f^NEO-FFI: NEO Five-Factor Inventory.

^g^STAI: State Trait Anxiety Inventory.

^h^WHOQOL-Bref: World Health Organization Quality of Life assessment instrument-Bref.

^i^QOL: quality of life.

##### Personality

Personality will be assessed using the NEO-FFI [35] and the STAI-Trait scale [36]. The 60-item NEO-FFI measures the Big Five personality domains: (1) Neuroticism, (2) Extraversion, (3) Openness to experience, (4) Agreeableness, and (5) Consciousness from the five factor model [35]. Each statement is rated on a five-point rating scale ranging from 1 (*strongly disagree*) to 5 (*strongly agree*), resulting in dimension scores between 12 and 60. The psychometrics has been extensively assessed and the internal consistency, test-retest reliability, and validity are acceptable to good [37].

The STAI (short form) consists of 20 items for measuring state anxiety (10 items) and trait anxiety (10 items) [36]. In this study, only the STAI-Trait scale will be used. This scale describes the person’s tendency to experience feelings of anxiety and stress. The STAI-Trait scale has a four-point rating scale ranging from 1 (*almost never*) to 4 (*almost always*). The Dutch version of the STAI is a reliable and valid instrument [38].

##### Quality of Life

QOL will be measured with the WHOQOL-Bref [39]. This 26-item questionnaire is a short version of the WHOQOL-100 and assesses four domains (Physical health, Psychological health, Social relationships, and Environment) as well as one general facet “Overall QOL and General Health”. The questions in the domains are derived from the 24 facets of the WHOQOL-100, with one item from each of the facets. Each item is rated on a five-point rating scale. Higher scores indicate better QOL [39,40]. The WHOQOL-Bref has good psychometric properties as prior research shows that the WHOQOL-Bref is a reliable and valid instrument [40-43].

##### Additional Assessments

Socio-demographic information (ie, sex, age, marital status, and education level) will be obtained from patients at baseline. Clinical information, including date of trauma treatment, ISS, type of trauma mechanism (eg, traffic accident or fall), type of injury (eg, fracture), trauma treatment (eg, operation or medication), consult or treatment from medical psychology (yes/no and which type of treatment), hospital stay (yes/no), in case of hospital stay, admission to intensive care unit, and duration of hospital stay will be abstracted from the patients’ medical records. Possible logistic problems will also be recorded.

### Statistical Analysis

#### Focus Groups

The recorded focus groups are analyzed using open, axial, and selective coding technique [26,27]. Open coding is used to identify different domains: physical, psychological, social, and environmental. Then, axial and selective coding is conducted to determine different themes. These codes consist of single words or short sentences. Two reviewers independently reviewed and coded each of the transcripts and ensured data saturation. Atlas.ti is used for analyzing the transcripts [44]. In addition, patient characteristics, PTSD, anxiety and depressive symptoms, and responses on the questionnaires were analyzed using descriptive statistics in SPSS version 22.

#### Observational Study

The patient characteristic will be studied using descriptive statistics. Then, the baseline characteristics (ie, sociodemographic, clinical, and psychological variables) of participants versus nonparticipants, participants who complete versus drop out during follow-up, and participants who are discharged versus being in the hospital after treatment in the shock room will be compared using independent t-tests and Chi-squared tests. Nonnormal continuous data will be analyzed with Mann-Whitney U tests or Fisher’s exact tests.

Repeated measures of latent class analysis will be used to analyze the courses (ie, time is independent variable) of ASD and PTSD (dependent variables). Moreover, to examine if these different courses of ASD and PTSD (independent variable) could be characterized by socio-demographic (eg, sex, age, education level, and living situation) and clinical (eg, type of trauma, ISS, Glasgow Coma Score, being hospitalized, being treated on the intensive care unit, complications during treatment, and treated by a medical psychologist or psychiatrist), and psychological (eg, anxiety, depressive symptoms, and personality) variables (dependent variables). As a result, each class will represent a different course of ASD and PTSD. By focusing on the characteristics of the different classes, a risk profile will, consequently, be developed to determine which patients are at risk for ASD or PTSD. Sociodemographic and clinical variables are examined as moderating effect, while psychological variables are studied as mediating effects.

Linear mixed models, repeated measures, will be used to examine the effect of ASD (independent variable) on PTSD, anxiety and depressive symptoms, and QOL domains (dependent variables) over time (see Table 2).

The ISS, type of injury and type of trauma mechanism (eg, traffic accident or fall) will be used as covariates. 

**Table 2:** Overview of statistical analysis

Baseline analysis and aims^a^	Independent variables	Dependent variables	Analyses
Patient characteristics	Sociodemographics	N/A^b^	Frequencies Descriptives
	Clinical variables	N/A	Frequencies Descriptives
	Psychological variables	N/A	Frequencies Descriptives
Comparison of patient characteristics	Participants versus nonparticipant	Sociodemographics Clinical variables Psychological variables	Continuous data: Independent t-test, Mann-Whitney U Categorical data: Chi-squared, Fishers’ exact test
	Completers versus noncompleters	Sociodemographics Clinical variables Psychological variables	Continuous data: Independent t-test, Mann-Whitney U Categorical data: Chi-squared, Fishers’ exact test
	Participants being discharged versus being in the hospital	Sociodemographics Clinical variables Psychological variables	Continuous data: Independent t-test, Mann-Whitney U Categorical data: Chi-squared, Fishers’ exact test
Aim 2: Course of ASD and PTSD	Time	ASD^c^ PTSD^d^	Repeated measures, latent class analysis
Aim 3: Risk profile	ASD PTSD	Sociodemographics Clinical variables Psychological	Repeated measures, latent class analysis
Aim 4: Effect of ASD	ASD	PTSD Anxiety Depressive symptoms QOL^e^	Linear Mixed models, repeated measures

^a^The dependent and independent variables for aim 1 could not be provided because this aim focuses on qualitative data.

^b^N/A: Not applicable.

^c^ASD: acute stress disorder.

^d^PTSD: posttraumatic stress disorder.

^e^QOL: quality of life.

## Results

Data collection and analysis for the focus group study are completed. Results will be reported in 2018. Enrollment of participants for the observational study began in November 2016. Data collection will be completed by the end of 2018. The study results will then be reported in 2019.

## Discussion

This is the first multi-method study in trauma patients that examines psychological consequences after injury, using both a qualitative focus group study design as well as an observational prospective design. In the focus group study, the aim was to interview patients about their experiences with the injury, treatment, and rehabilitation. Since it is unknown if and in what way patients’ experiences contribute to the development of psychological problems and disorders. The observational study will examine the course of ASD and PTSD, as ASD after injury is less studied and it is unknown how ASD and PTSD develop over time up to 12 months after injury. Moreover, as a result of all outcome measures, a risk profile of patients may be determined to predict which patients are at risk for developing ASD or PTSD. Altogether, this study will provide information concerning which patients to include in further research that focuses on psychological intervention.

Several factors related to the design and execution must be taken into account. First, response bias may occur in the focus group study. Patients may decline participation because they are not interested in discussing their experiences, or it might be too confronting to talk about their experiences and psychological problems. Second, it is known that the population of trauma patients has a broad variety of trauma mechanisms and injuries. Therefore, it might be difficult to generalize to the whole trauma population. However, concerning the observational study, almost all trauma patients being treated in the shock room will be included from two different Level-1 trauma centers so data saturation can be reached. These centers are located in different provinces and cities in The Netherlands. Therefore, a representative population in the observational study can be included. Third, patients with severe injuries might be less capable to complete the baseline questionnaire almost directly after injury due to being treated at the intensive care unit. Patients will, therefore, be asked to fill in the date of completing the questionnaire and if they needed any help. Then, the time between injury and measurement can be analyzed. This provides information on what time severely injured patients are capable to complete the baseline questionnaire.

In conclusion, this study is exploratory in nature and it will contribute to the need for information on psychological consequences after injury. Then, awareness in clinicians about the consequences can be created so they are able to act more effective and patient-oriented care can be provided.

## Appendix

Multimedia Appendix 1 DSM-5 Diagnostic criteria for acute stress disorder (ASD).

Multimedia Appendix 2 DSM-5 Diagnostic criteria for posttraumatic stress disorder (PTSD).

## Abbreviations

ASD: acute stress disorder
DSM: Diagnostic and Statistical Manual of Mental Disorders
HADS: Hospital Anxiety and Depression Scale
IES-R: Impact of Event Scale-Revised
ISS: Injury Severity Score
NEO-FFI: NEO Five-Factor Inventory
PTSD: posttraumatic stress disorder
QOL: quality of life
STAI: State Trait Anxiety Inventory
WHOQOL-Bref: World Health Organization Quality of Life assessment instrument-Bref

## References

[1] Centraal Bureau voor de Statistiek (2017). (Archived by WebCite® at Published-09-20.

[2] Meerding WJ, Mulder S, van BEF (2006). Incidence and costs of injuries in The Netherlands. Eur J Public Health.

[3] Lansink KWW, Gunning AC, Spijkers ATE, Leenen LPH (2013). Evaluation of trauma care in a mature level I trauma center in the Netherlands: outcomes in a Dutch mature level I trauma center. World J Surg.

[4] Pino SFI, Ballesteros SMA, Cordero LL, Guerrero LF, TraumaNeurointensive Care Work Group of the SEMICYUC (2015). Quality of trauma care and trauma registries. Med Intensiva.

[5] Nijboer JMM, van DSCK, van DNJ, Nijsten MWN, Ten DH (2007). Two cohorts of severely injured trauma patients, nearly two decades apart: unchanged mortality but improved quality of life despite higher age. J Trauma.

[6] de Jongh MA, Meeuwis JD, van Baar ME, van Stel HF, Schrijvers AJ (2008). Evaluation of trauma care by comparing mortality risks and admission policy in a Dutch trauma region. Injury.

[7] Gouin J, Kiecolt-Glaser JK (2011). The impact of psychological stress on wound healing: methods and mechanisms. Immunol Allergy Clin North Am.

[8] Visser E, Gosens T, Den Oudsten BL, De Vries J (2017). The course, prediction and treatment of acute and post-traumatic stress in trauma patients: A systematic review. J Trauma Acute Care Surg.

[9] Clay FJ, Newstead SV, Watson WL, McClure RJ (2010). Determinants of return to work following non life threatening acute orthopaedic trauma: a prospective cohort study. J Rehabil Med.

[10] Clay FJ, Newstead SV, Watson WL, Ozanne-Smith J, Guy J, McClure RJ (2010). Bio-psychosocial determinants of persistent pain 6 months after non-life-threatening acute orthopaedic trauma. J Pain.

[11] Archer KR, Castillo RC, Wegener ST, Abraham CM, Obremskey WT (2012). Pain and satisfaction in hospitalized trauma patients: the importance of self-efficacy and psychological distress. J Trauma Acute Care Surg.

[12] Wilson K, von der Heyde R, Sparks M, Hammerschmidt K, Pleimann D, Ranz E, Rector J, Sniezak D (2014). The impact of demographic factors and comorbidities on distal radius fracture outcomes. Hand (N Y).

[13] Nota SPFT, Bot AGJ, Ring D, Kloen P (2015). Disability and depression after orthopaedic trauma. Injury.

[14] van Delft-Schreurs CC, van Bergen JJ, de Jongh MA, van de Sande P, Verhofstad MHJ, De Vries J (2014). Quality of life in severely injured patients depends on psychosocial factors rather than on severity or type of injury. Injury.

[15] Bhandari M, Busse JW, Hanson BP, Leece P, Ayeni OR, Schemitsch EH (2008). Psychological distress and quality of life after orthopedic trauma: an observational study. Can J Surg.

[16] Crichlow RJ, Andres PL, Morrison SM, Haley SM, Vrahas MS (2006). Depression in orthopaedic trauma patients. Prevalence and severity. J Bone Joint Surg Am.

[17] Holbrook TL, Anderson JP, Sieber WJ, Browner D, Hoyt DB (1999). Outcome after major trauma: 12-month and 18-month follow-up results from the Trauma Recovery Project. J Trauma.

[18] Holbrook TL, Hoyt DB (2004). The impact of major trauma: quality-of-life outcomes are worse in women than in men, independent of mechanism and injury severity. J Trauma.

[19] Michaels AJ, Michaels CE, Smith JS, Moon CH, Peterson C, Long WB (2000). Outcome from injury: general health, work status, and satisfaction 12 months after trauma. J Trauma.

[20] Rusch MD (1998). Psychological response to trauma. Plast Surg Nurs.

[21] O'Donnell ML, Creamer M, Elliott P, Atkin C, Kossmann T (2005). Determinants of quality of life and role-related disability after injury: impact of acute psychological responses. J Trauma.

[22] American Psychiatric Association (2014). Handboek voor de classificatie van psychische stoornissen (DSM-5). Uitgeverij Boom.

[23] Comer R (2013). Abnormal psychology.

[24] Bryant RA, O'Donnell ML, Creamer M, McFarlane AC, Silove D (2013). A multisite analysis of the fluctuating course of posttraumatic stress disorder. JAMA Psychiatry.

[25] Osenbach JE, Lewis C, Rosenfeld B, Russo J, Ingraham LM, Peterson R, Wang J, Zatzick DF (2014). Exploring the longitudinal trajectories of posttraumatic stress disorder in injured trauma survivors. Psychiatry.

[26] Krueger R, Casey M (2014). Focus groups. A practical guide for applied research. 5th Edition.

[27] Boeije H (2008). Analyseren in kwalitatief onderzoek.

[28] Barbour RS, Morgan DL (2017). A new era in focus group research: Challenges, Innovation and Practice.

[29] Bonate PL (2001). A brief introduction to Monte Carlo simulation. Clin Pharmacokinet.

[30] Brom D, Kleber RJ (1985). De Schok Verwerkings Lijst (SVL). Nederlands Tijdschrift voor de Psychologie.

[31] Wohlfarth T.D., van den Brink W, Winkel F.W., ter Smitten M (2003). Screening for posttraumatic stress disorder: An evaluation of two self-report scales among crime victims. Psychological Assessment.

[32] Creamer M, Bell R, Failla S (2003). Psychometric properties of the Impact of Event Scale - Revised. Behav Res Ther.

[33] Van Der Ploeg E, Mooren T, Kleber R.K., Van Der Velden P.G., Brom D. (2004). Internal validation of the dutch version of the impact of event scale. Psychological Assessment.

[34] Zigmond AS, Snaith RP (1983). The hospital anxiety and depression scale. Acta Psychiatr Scand.

[35] Costa PT, McCrae RR (1992). Revised NEO personality inventory (NEO-PI-R) and NEO five factor inventory (NEO-FFI) professional manual.

[36] Spielberger CD, Gorsuch RL, Lushene RE (1970). STAI manual for the state-trait anxiety inventory.

[37] Hoekstra H, Ormel J, De Fruyt F (2007). Handleiding NEO-PI-R en NEO-FFI persoonlijkheidsvragenlijsten.

[38] De Vries J, Van Heck GL (2013). Development of a short version of the Dutch version of the Spielberger STAI trait anxiety scale in women suspected of breast cancer and breast cancer survivors. J Clin Psychol Med Settings.

[39] The World Health Organization Quality of Life Group (1998). Development of the world health organization WHOQOL-BREF quality of life assessment. Psychological Medicine.

[40] The World Health Organization Quality of Life Group (1998). The world health organization quality of life assessment (WHOQOL): Development and general psychometric properties. Social Science & Medicine.

[41] Taylor WJ, Myers J, Simpson RT, McPherson KM, Weatherall M (2004). Quality of life of people with rheumatoid arthritis as measured by the World Health Organization Quality of Life Instrument, short form (WHOQOL-BREF): score distributions and psychometric properties. Arthritis Rheum.

[42] Skevington SM, Lotfy M, O'Connell KA (2004). The World Health Organization's WHOQOL-BREF quality of life assessment: psychometric properties and results of the international field trial. A report from the WHOQOL group. Qual Life Res.

[43] Van Esch L, Den Oudsten BL, De Vries J (2011). The world health organization quality of life instrument-short form (WHOQOL-BREF) in women with breast problems. International Journal of Clinical and Health Psychology.

[44] Friese S (2014). Qualitative data analysis with ATLAS ti.

