# Supplementary material for: Physical Trauma Patients with Symptoms of an Acute and Posttraumatic Stress Disorder: Protocol for an Observational Prospective Cohort Study
Source: JMIR Res Protoc. 2018 Mar 29;7(3):e88. doi: 10.2196/resprot.9006 (PMC5897623; doi:10.2196/resprot.9006)
Supplement: Multimedia Appendix 1 [file resprot_v7i3e88_app1.pdf]

A.

Exposure to actual or threatened death, serious injury, or sexual violation in one (or more) of the following ways:

1. Directly experiencing the traumatic event(s).
2. Witnessing, in person, the event(s) as it occurred to others.
3. Learning that the event(s) occurred to a close family member or close friend
   Note: In cases of actual or threatened death of a family member or friend, the event(s) must have been violent or accidental.
4. Experiencing repeated or extreme exposure to aversive details of the traumatic event(s) (e.g., first responders collecting human remains; police officers repeatedly exposed to details of child abuse)
   Note: Criterion A4 does not apply to exposure through electronic media, television, movies, or pictures, unless this exposure is work related.

B.

Presence of nine (or more) of the following symptoms from any of the five categories of intrusion, negative mood, dissociation, avoidance, and arousal, beginning or worsening after the traumatic event(s) occurred:

1. Recurrent, involuntary, and intrusive distressing memories of the traumatic event(s).
   Note: In children, repetitive play may occur in which themes or aspects of the traumatic event(s) are expressed.
2. Recurrent distressing dreams in which the content and/or affect the dram are related to the event(s).
   Note: In children, there may be frightening dreams without recognizable content.
3. Dissociative reactions (e.g., flashbacks) in which the individual feels or acts as if the traumatic event(s) were recurring. (Such reactions may occur on a continuum, with the most extreme expression being a complete loss of awareness of present surroundings.)
   Note: In young children, trauma-specific reenactment may occur in play.
4. Intense or prolonged psychological distress at exposure to internal or external cues that symbolize or resemble an aspect of the traumatic event(s).

Negative mood

5. Persistent inability to experience positive emotions (e.g., inability to experience happiness, satisfaction, or loving feelings).

Dissociative Symptoms

6. An altered sense of reality of one’s surroundings or oneself (e.g., seeing oneself from another’s perspective, being a daze, time slowing).
7. Inability to remember an important aspect of the traumatic event(s) (typically due to dissociative amnesia and not to other factors such as head injury, alcohol, or drugs).

Avoidance Symptoms

8. Efforts to avoid distressing memories, thoughts, or feelings about or closely associated with the traumatic event(s).
9. Efforts to avoid external reminders (people, places, conversations, activities,

objects, situations) that arouse distressing memories, thoughts, or feelings about closely associated with the traumatic event(s).

Arousal Symptoms

10. Sleep disturbance (e.g., difficulty falling or staying asleep, restless sleep).
11. Irritability and angry outbursts (with little or no provocation) typically expressed as verbal or physical aggression toward people or objects.
12. Hypervigilance
13. Problems with concentration
14. Exaggerated startle response.

C. Duration of the disturbance (symptoms in Criteria B) is 3 days to 1 month after trauma exposure.
Note: symptoms typically begin immediately after the trauma, but persistence for at least 3 days and up to a month are needed to meet disorder criteria.

D. The disturbance causes clinically significant distress or impairment in social, occupational, or other important areas of functioning.

E. The disturbance is not attributable to the physiological effects of a substance (e.g., medication or alcohol) or another medical condition (e.g., mild traumatic brain injury) and is not better explained by brief psychotic disorder.
