# Supplementary material for: Physical Trauma Patients with Symptoms of an Acute and Posttraumatic Stress Disorder: Protocol for an Observational Prospective Cohort Study
Source: JMIR Res Protoc. 2018 Mar 29;7(3):e88. doi: 10.2196/resprot.9006 (PMC5897623; doi:10.2196/resprot.9006)
Supplement: Multimedia Appendix 2 [file resprot_v7i3e88_app2.pdf]

**A.**

Exposure to actual or threatened death, serious injury, or sexual violence in one (or more) of the following ways:

1. Directly experiencing the traumatic event(s).
2. Witnessing, in person, the event(s) as it occurred to others.
3. Learning that the traumatic event(s) as it occurred to a close family member or close friend. In cases of actual or threatened death of a family member or friend, the event(s) must have been violent or accidental.
4. Experiencing repeated or extreme exposure to aversive details of the traumatic event(s) (e.g., first responders collecting human remains; police officers repeatedly exposed to details of child abuse)
   Note: Criterion A4 does not apply to exposure through electronic media, television, movies, or pictures, unless this exposure is work related.

**B.**

Presence of one (or more) of the following intrusion symptoms associated with the traumatic event(s), beginning after the traumatic event(s) occurred:

1. Recurrent, involuntary, and intrusive distressing memories of the traumatic event(s),
   Note: In children older than 6 years, repetitive play may occur in which themes or aspects of the traumatic event(s) are expressed.
2. Recurrent distressing dreams in which the content and/or affect of the dream are related to traumatic event(s).
   Note: In children, there may be frightening dreams without recognizable content.
3. Dissociative reactions (e.g., flashbacks) in which the individual feels or acts as if the traumatic event(s) were recurring. (Such reactions may occur on a continuum, with the most extreme expression being a complete loss of awareness of present surroundings.)
   Note: In young children, trauma-specific reenactment may occur in play.
4. Intense or prolonged psychological distress at exposure to internal or external cues that symbolize or resemble an aspect of the traumatic event(s).
5. Marked physiological reactions to internal or external cues that symbolize or resemble an aspect of the traumatic event.

**C.**

Persistent avoidance of stimuli associated with the traumatic event(s), beginning after the traumatic event(s) occurred, as evidenced by one or both of the following:

1. Avoidance of or efforts to avoid distressing memories, thoughts, or feelings, about or closely associated with the traumatic event(s)
2. Avoidance of or efforts to avoid external reminders (people, places, conversations, activities, objects, situations) that arouse distressing memories, thoughts, or feelings about or closely associated with the traumatic event(s)

**D.**

Negative alterations in cognitions and mood associated with the traumatic event(s), beginning or worsening after the traumatic event(s) occurred, as evidenced by two (or more) of the following:

1. Inability to remember an important aspect of the traumatic event(s) (typically due

to dissociative amnesia and not to other factors such as head injury, alcohol, or drugs).
2. Persistent and exaggerated negative beliefs or expectations about oneself, others, or the world (e.g., “I am bad,” “No one can be trusted,” “The world is completely dangerous,” “My whole nervous system is permanently ruined.”).
3. Persistent, distorted cognitions about the cause or consequences of the traumatic event(s) that lead the individual to blame himself/herself or others.
4. Persistent negative emotional state (e.g., fear, horror, anger, guilt, or shame).
5. Markedly diminished interest or participation in significant activities.
6. Feelings of detachment or estrangement from others.
7. Persistent inability to experience positive emotions (e.g., inability to experience happiness, satisfaction, or loving feelings).

**E.**

Marked alternations in arousal and reactivity associated with the traumatic event(s), beginning or worsening after the traumatic event(s) occurred, as evidenced by two (or more) of the following:

1. Irritability and angry outbursts (with little or no provocation) typically expressed as verbal or physical aggression toward people or objects.
2. Reckless or self-destructive behaviour
3. Hypervigilance.
4. Exaggerated startle response
5. Problems with concentration
6. Sleep disturbance (e.g., difficulty falling or staying asleep or restless sleep).

**F.**

Duration of the disturbance (symptoms in Criteria B, C, D, and E) is more than 1 month.

**G.**

The disturbance causes clinically significant distress or impairment in social, occupational, or other important areas of functioning.

**H.**

The disturbance is not attributable to the physiological effects of substance (e.g., medication, alcohol) or another medical condition.

*Specify* whether:

With dissociative symptoms: The individual’s symptoms meet the criteria for posttraumatic stress disorder, and in addition, in response to the stressor, the individual experiences persistent or recurrent symptoms of the following:

1. Depersonalization: Persistent or recurrent experiences of feeling detached from, and as if one were an outside observer of, one’s mental processes or body (e.g., the world around the individual is experienced as unreal, dreamlike, distant, or distorted.
2. Derealization: Persistent or recurrent experiences of unreality of surroundings (e.g., the world around the individual is experienced as unreal, dreamlike, distant, or distorted).

Note: To use this subtype, the dissociative symptoms must not be attributable to the physiological effects of a substance (e.g., blackouts, behavior during alcohol intoxication) or another medical condition (e.g., complex partial seizures).

*Specify* if:

With delayed expression: If the full diagnostic criteria are not met until at least 6 months after the event (although the onset and expression of some symptoms may be immediate).
